# Gene regulatory networks and their applications: understanding biological and medical problems in terms of networks

**DOI:** 10.3389/fcell.2014.00038

**Published:** 2014-08-19

**Authors:** Frank Emmert-Streib, Matthias Dehmer, Benjamin Haibe-Kains

**Affiliations:** ^1^Computational Biology and Machine Learning Laboratory, Faculty of Medicine, Health and Life Sciences, Center for Cancer Research and Cell Biology, School of Medicine, Dentistry and Biomedical Sciences, Queen's University BelfastBelfast, UK; ^2^Institute for Bioinformatics and Translational Research, UMITHall in Tyrol, Austria; ^3^Bioinformatics and Computational Genomics Laboratory, Department of Medical Biophysics, Princess Margaret Cancer Centre, University of TorontoCanada

**Keywords:** gene regulatory networks, computational genomics, statistical inference, network analysis, biomarker, personalized medicine, systems biology

## Abstract

In recent years gene regulatory networks (GRNs) have attracted a lot of interest and many methods have been introduced for their statistical inference from gene expression data. However, despite their popularity, GRNs are widely misunderstood. For this reason, we provide in this paper a general discussion and perspective of gene regulatory networks. Specifically, we discuss their meaning, the consistency among different network inference methods, ensemble methods, the assessment of GRNs, the estimated number of existing GRNs and their usage in different application domains. Furthermore, we discuss open questions and necessary steps in order to utilize gene regulatory networks in a clinical context and for personalized medicine.

## 1. Introduction

About 15 years ago inference of large-scale gene regulatory networks (GRNs) was made possible thanks to the availability of high-throughput gene expression data. Within this time, many different methods have been developed (Liang et al., 1998; Butte et al., 2000; Friedman, 2004; Wille et al., 2004; Zhang et al., 2011) and used to enhance our understanding of diseases (Basso et al., 2005; Madhamshettiwar et al., 2012). However, despite their widespread usage in current biomedical research, there is still much confusion about the basic meaning of GRNs, ways of assessment, and possible application areas.

In this paper, we aim to clarify some of these problems and also provide a discussion of important next steps in order to bring gene regulatory networks closer to the clinical and medical application. Furthermore, we add some recommendations we consider important to make GRNs more popular among biologists and clinicians, as they require a dedicated platform for accessing and analyzing inferred gene regulatory networks.

## 2. How do we call networks inferred from gene expression data

For reasons of clarity, we first define what we mean by a *gene regulatory network*.

**Definition 1**. *We call a network that has been inferred from gene expression data a “gene regulatory network,” briefly denoted as GRN*.

From the above definition one can see that we are assuming a statistical perspective placing the data in the center of focus. Due to the nature of gene expression data, providing information about the abundance of mRNAs only rather than binding information, gene regulatory networks defined in the above sense provide information about regulatory interactions between regulators and their potential targets; gene-gene interactions, and potential protein-protein interactions (e.g., in a complex) (de Matos Simoes et al., 2013a).

There are many examples where such networks have been studied (Margolin et al., 2006; Werhli et al., 2006; Meyer et al., 2008; Stolovitzky et al., 2009; Emmert-Streib et al., 2012); see Table 1 for a brief overview. So far there is no generally adopted parlance to name such inferred networks, but the term *gene regulatory network* (Hecker et al., 2009) is frequently used and will also be utilized in this paper.

**Table 1:** **Brief overview of statistical network inference methods that have been introduced in recent years and the key methods (second column) on which the inference algorithms are based on to estimate interactions**.

**Name**	**Method**	**References**
RN	Mutual information	Butte and Kohane, 2000
Aracne	Mutual information, DPI	Margolin et al., 2006
CLR	Mutual information with background	Faith et al., 2007
C3Net	Maximal mutual information	Altay and Emmert-Streib, 2010a
BC3Net	Bagging C3Net	de Matos Simoes and Emmert-Streib, 2012
GENIE3	Regression	Huynh-Thu et al., 2010
GGM	Full partial correlation	Wille et al., 2004
MRNet	Conditional mutual information	Meyer et al., 2008
MI3	Three-way mutual information	Luo et al., 2008

For completeness, we would like to mention that there are a variety of conceptually different approaches to infer networks and we would like to refer the reader to the review articles by Lee et al. (2009); Markowetz et al. (2007) for a thorough discussion.

## 3. Is there just one “right” method?

In the last years, there have been many network inference methods introduced and many comparisons have been conducted (Akutsu et al., 1999; Margolin et al., 2006; Werhli et al., 2006; Meyer et al., 2008; Stolovitzky et al., 2009; Emmert-Streib et al., 2012). As it seems, the results of such technical comparisons depend crucially on the studied conditions, including; type of the data (simulated, real), size of the network, number of samples, amount of noise, experimental design (observational, experimental, interventional), type of the underlying interaction structure (scale-free, random, small-world), error measure (global, local), among others. For this reason it is unlikely that there is one “right” method that fits all different biological, technical and experimental design conditions best.

However, if one asks less technical and more biological questions about the meaning of the inferred networks, i.e., by evaluating the biological consistency of inferred networks resulting from different network inference methods, there is supporting evidence that the differences might not be that large, as recently demonstrated for C3Net, BC3Net and Aracne (de Matos Simoes et al., 2013b). Hence, it is unlikely that there is just one method that outperforms all others for all conditions, but a number of methods result in an overlapping spectrum having the potential to infer similar biological information.

## 4. Ensemble methods

A recent trend in the field of biological network inference is the use of *ensemble methods* (Zhang and Singer, 2010) to improve their stability and accuracy. Ensemble methods have been popularized by Leo Breiman as exemplified by random forest classifiers (Breiman, 2001) that have at their heart *bagging* (Breiman, 1996). Briefly, the underlying idea is to (1) bootstrap a given data set, (2) apply a network inference method, and (3) aggregate all separate outcomes into a final result. Here, it is possible to apply for each bootstrap data set the same inference method or different methods, leading to the distinction between homogeneous and heterogeneous ensemble methods. Examples for network inference methods that are based on this principle are (Huynh-Thu et al., 2010; de Matos Simoes and Emmert-Streib, 2012; Marbach et al., 2012).

Although ensemble approaches to network inference are computationally intensive, they have the clear advantage of being straightforwardly and efficiently implemented in large computer cluster. Indeed, if one runs an ensemble of size *B* on a computer cluster with *B* nodes, the computation time for the whole ensemble is (about) the same as for just one method run on one desktop computer.

## 5. Assessing inferred networks

The assessment of inferred networks is an important and complicated topic. The reason for this is that networks are high-dimensional, structured objects that enable modeling of diverse aspects of biological systems. There are two main issues one has to face when assessing the quality of inferred biological networks: (I) the definition of a set of “true” interactions, referred to as gold standard and (II) the choice of statistical measures to quantitatively assess the quality of networks using this gold standard. The former issue is usually addressed by using known interactions from research articles (Mostafavi et al., 2008; Haibe-Kains et al., 2012) and structured biological databases such as KEGG (Kanehisa and Goto, 2000) or I2D (Brown and Jurisica, 2005). The main disadvantage of this approach is that, although the set of known interactions might be quite large, many of them might not be relevant to the biological conditions under investigation. For this reason, it is also important to note that the standardized reporting of such contextual information is crucial for comparing causal and correlative relationships between molecular entities meaningfully. Examples for such are endeavors that provide computer processable languages are BEL, PySB, and BCML (Slater, 2014).

As an alternative, several research groups performed multiple perturbations of the biological system under study (cancer cell lines for instance) to measure their effects and subsequently validate their inferred networks (Frohlich et al., 2008; Olsen et al., 2014). This experimental design, although significantly more lengthy and costly, enables to validate inferred interactions in conditions that are identical or closely mimic those used for network inference. As an example, Olsen et al. knocked down 8 genes in the RAS signaling pathway in colorectal cancer cell lines to quantitatively assess the quality of gene interaction networks built from expression data of human colon tumors.

Given a set of known interactions, one can use traditional statistical error measures, such as F-score or AUC-ROC (area under the receiver operating characteristics curve). These measures can be used to assess the quality of networks at the global-level (for the network as a whole) or at the edge-level (for each individual edge) or for many intermediate-levels (for instance for network-motifs); see Altay and Emmert-Streib (2010b); Emmert-Streib and Altay (2010). That means, already for generic statistical error measures there are many different levels that can be assessed. Furthermore, real biological data and simulated data can, and should, be used for the assessment of networks. For real biological data this allows to assess the biological relevance of inferred networks, e.g., by using GO or KEGG, and simulated data enable a detailed analysis of any technical aspect. In general, one should use a large variety of quality and error measures on a routine way. Unfortunately, standards are currently not available, but would need to be developed.

## 6. How many gene regulatory networks exist?

**Figure 1:**
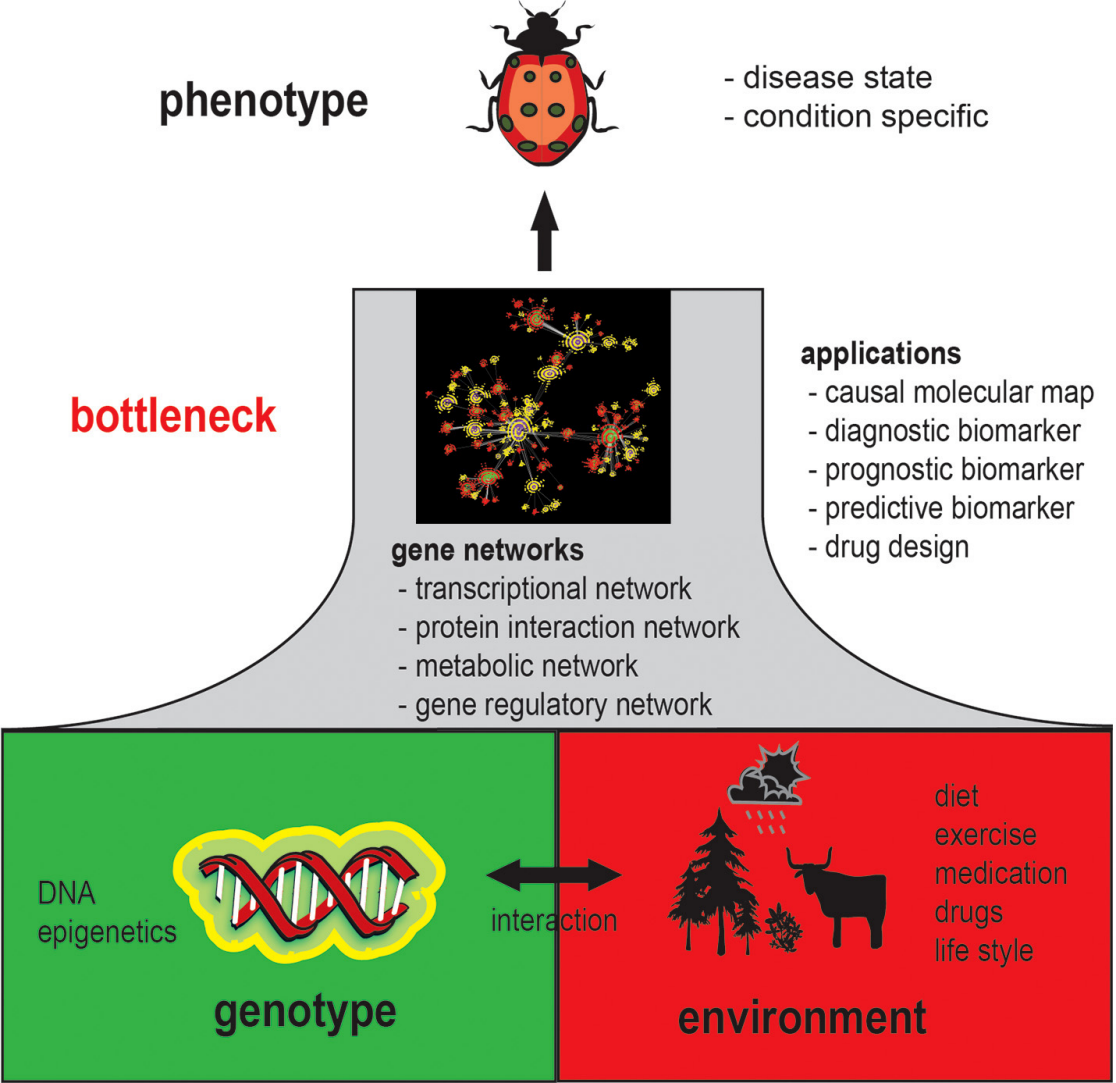
**Schematic overview of the general role gene networks play in understanding phenotypes**.

It is generally acknowledged that a phenotype is an emergent property of genotype-environment interactions. Specifically, a phenotype results from molecular and cellular activity patterns from genotype-environment interactions. This implies that each observable phenotype is associated with phenotype-specific gene networks, because without changing molecular interactions a phenotype cannot change; this concept is illustrated in Figure 1. In this figure, gene networks can be seen as a bottleneck between the genotype and the phenotype with respect to their coupling. That means every change on the genotype level that will result in a change of the phenotype will also inevitably lead to a change in the gene network structure as mediator between both levels.

However, since gene networks refer to all possible types of molecular networks, including the transcriptional regulatory network, protein interaction network, metabolic network, gene regulatory network and interactions between these networks, it is less clear which of these networks, or all of them, are actually changed. Moreover, because a gene regulatory network can potentially represent many types of physical biochemical interactions among genes and gene products (de Matos Simoes et al., 2013a) it can be expected that gene regulatory networks are highly phenotype specific (Schadt, 2009; Emmert-Streib and Glazko, 2011). Establishing such relationships will therefore be a complex task, but also provides an opportunity to catalog phenotypes quantitatively. An example for the analysis of tissue-specific networks can be found in Guan et al. (2012) where 107 tissue specific network have been studied. Currently, the number of GRNs is difficult to estimate but based on these preliminary results one can hypothesize that there are more than 200 different GRNs for Human alone, because this corresponds about to the number of different cell types. However, also pathological cells manifesting tumors have their own characteristic networks (Emmert-Streib et al., 2014) implying that there are probably thousands of different gene networks in Human.

## 7. Usage of gene regulatory networks

It is important to emphasize that the inference of gene regulatory networks is not the final result, but these networks are supposed to help in solving a number of different biological and biomedical problems.

### 7.1. Causal map of molecular interactions

Maybe the most frequently named use of gene regulatory networks is to serve as a “map” or a “blueprint” of molecular interactions. In this respect such a network can be used to derive novel biological hypothesis about molecular interactions, e.g., for the transcription regulation of genes, which can then be investigated in wet lab experiments by using, e.g., ChIP-chip and gene expression experiments (Bussemaker et al., 2001; Basso et al., 2005). In such a case GRNs represent causal biochemical interactions because the predicted links are supposed to correspond to actual physical binding events between molecules. It is important to note that the inference of such causal interactions between gene products is a challenging task, because it goes beyond the mere association between such entities that would include also indirect relations/interactions involving intermediate gene products as well. However, despite the limitations of association networks it is interesting to note that also such networks capture valuable biological information (Butte et al., 2000).

An important aspect of this application is that the GRNs represent statistically significant predictions of molecular interactions obtained from large-scale data. Given the very large number of potential interactions between ~20,000 genes in Human and ~6000 gene in yeast, the GRNs are of tremendous help in narrowing these numbers down to potential interactions for which statistical support is available. Overall, this enables more effective experiments by an adopted experimental design.

### 7.2 Experimental design and perturbation experiments

An under appreciated applicability of gene regulatory networks is to use these for guiding the experimental design of new experiments. Specifically, many high-throughput experiments are screening experiments generating observational data. That means, these experiments are not controlled by establishing conditions that enhance molecular target processes to improve the signal strength of these, but they merely “observe” the state of the systems as it is, without interventions or perturbations. A downside of such screening experiments is that the signal about, e.g., certain pathways, may be too low to be inferable by statistical means. However, using prior knowledge about “partial” gene regulatory networks inferred from such observational data may allow to overcome these obstacles systematically and help in designing perturbation or intervention experiments to stimulate the molecular system purposefully. That means by identifying the parts of the molecular system that are not well detected, based on inferred networks, dedicated perturbations can be constructed to boost their active responses.

### 7.3. Networks as biomarkers

In recent studies, it has been argued that (sub-)networks could also be used as biomarkers, e.g., for diagnostic, predictive or prognostic purposes (Chuang et al., 2007; Ben-Hamo and Efroni, 2011; Chen et al., 2011; Dehmer et al., 2013a). This is particularly plausible for a complex disorder like cancer, because the hallmarks of cancer are represented by pathways rather than individual genes (Hanahan and Weinberg, 2011) and the crucial aspect of pathways is that their constituting genes are actively interacting with each other. For this reason, network-based biomarkers can be seen as statistical measures that consider the interaction structure between individual genes explicitly. In contrast, biomarkers based on individual genes neglect these completely. For further applications of network-based biomarkers see also Dehmer et al. (2013b).

In the near future, we expect to see similar applications also for other types of complex disorders, because, despite their differences among each other, all of them share a need for considering interaction changes. Unfortunately, developing network-based biomarkers is considerably more complex than using univariate and multivariate gene signatures. Also, quantitatively, it remains to date unclear which gain one should expect from this new type of biomarkers, if any Staiger et al. (2012).

### 7.4. Comparative network analysis

When more and more gene regulatory networks from different physiological and disease conditions become available, it will be possible to statistically compare these networks (Dehmer and Emmert-Streib, 2007; Dehmer and Mehler, 2007). This will allow to learn about interaction changes across different physiological or disease conditions and enrich our biological and biomedical understanding of such phenotypes (Ideker and Krogan, 2012; Islam et al., 2013). Besides using classical comparative measures such as the *graph edit distance* or the Zelinka distance, topological indices could be also employed for such an analysis, see Dehmer et al. (2013c). It might be challenging to determine which similarity or distance measures are suitable to perform such a comparative network analysis and different types of networks as well as different biological questions may require different approaches, see (Sharan and Ideker, 2006; Przulj, 2007; Mithani et al., 2011; Pache and Aloy, 2012) for protein interaction networks or metabolic networks, for instance.

However, in order for this approach to succeed it will be necessary to establish databases, similar to sequence or protein structure databases, that provide free access to the inferred gene regulatory networks from different physiological and disease conditions. To this end, it may be necessary to form an international coalition because the expected effort to establish such a database and interactive access interfaces is anticipated to be larger than of sequence databases.

### 7.5. Network medicine and drug design

For establishing a network medicine useful for clinicians, it will be necessary to integrate different types of gene networks with each other (Shapira et al., 2009; Barabási et al., 2011), because each network type carries information about particular molecular aspects. For example, whereas the transcriptional regulatory network contains only information about the controlling regulations of gene expression, protein interaction networks represent information about protein-protein complexes. Taken together, an integration of various important molecular interaction types results in a comprehensive overview of regulatory programs and organizational architectures. Also, information about temporal changes in the network structure are important to understand immune response, infection and differentiation processes (Rozenblatt-Rosen et al., 2012; Yosef et al., 2013).

Also for a more efficient design of rational drugs the utilization of gene networks are indispensable (Ghosh and Basu, 2012; Fortney et al., 2013). For this reason, both subjects would profit tremendously if there would be more large-scale gene expression data available together with, e.g., survival data and drug-dose response information. This would allow to create, e.g., a connectivity map (Lamb et al., 2006) that is based on the similarity of molecular interaction networks rather than on the mere similarity of expression profiles. Overall, this would help us on our way to a more personalized medicine (Chan and Ginsburg, 2011), because condition specific gene regulatory networks are *closer* to the phenotype than genetic or epigenetic markers; see Figure 1 for a visualization.

## 8. Knowledge platform for medical and clinical practice

It is important to emphasize that gene regulatory networks are not the final outcome of a biological or biomedical study, but an intermediate result. For this reason, interrogation platforms are needed that allow the downstream analysis of such networks. Specifically, aside from databases that store inferred GRNs, network analysis tools and visualization layouts are needed that allow an easy integration with biological and clinical information, e.g., in form of GO and KEGG databases or clinical patient and general epidemiological data. Such a knowledge platform should hold cross-disease information similar to the OMIM (Online Mendelian Inheritance in Man).

Furthermore, it would be desirable if such a knowledge platform has an intuitive to use interface allowing also non-technical experts the exploration of GRNs. For a practical realization, e.g., the tranSMART platform (Athey et al., 2013) could be utilized. TranSMART is based on the open source i2b2 (informatics for integrating biology and the bedside) framework, sponsored by the NIH Roadmap NCBC, to provide clinicians with the tools to integrate clinical genomics with medical patient record data. An attractive feature of tranSMART is that it can be combined with Galaxy, an open, web-based user interface, which allows the connection to a variety of programming languages. That means a researcher without specific bioinformatic expertise can utilize R scripts, e.g., provided by Bioconductor, CRAN or individually developed packages, via a web-based graphical user interface for analyzing GRNs. Importantly, Galaxy offers also several mechanisms to ensure the reproducibility of research results.

## 9. Conclusion

In this paper, we discussed important aspects of gene regulatory networks inferred from gene expression data. Due to the multifaceted nature of GRNs, for which we gave some examples in this paper, a discussion about these networks cannot be one-dimensional because this would give a misleading impression of their meaning and potential usage. For this reason, we tried to provide a broad discussion touching upon a variety of different aspects to emphasize the intriguing depth offered by gene regulatory networks.

We think that neither the future of biology nor medicine is conceivable without gene networks in general, whereas gene regulatory networks form an important subtype of these, because such networks can be seen as a practical embodiment of systems biology. However, in order to exploit and utilize such networks efficiently in molecular biology, cellular biology and the biomedical sciences we need to establish comprehensive databases.

## Funding

Matthias Dehmer thanks the Austrian Science Funds for supporting this work (project P26142). Matthias Dehmer also gratefully acknowledges funding from the Standortagentur Tirol (formerly Tiroler Zukunftsstiftung).

### Conflict of interest statement

The authors declare that the research was conducted in the absence of any commercial or financial relationships that could be construed as a potential conflict of interest.

## References

[B1] Akutsu T. Miyano S. Kuhara S. (1999). Identification of genetic networks from a small number of gene expression patterns under the Boolean network model. Pacif. Symp. Biocomput. 17–28. 10380182 10.1142/9789814447300_0003

[B2] Altay G. Emmert-Streib F. (2010a). Inferring the conservative causal core of gene regulatory networks. BMC Syst. Biol. 4:132. 10.1186/1752-0509-4-132 20920161 PMC2955605

[B3] Altay G. Emmert-Streib F. (2010b). Revealing differences in gene network inference algorithms on the network-level by ensemble methods. Bioinformatics 26, 1738–1744. 10.1093/bioinformatics/btq259 20501553

[B4] Athey B. D. Braxenthaler M. Haas M. Guo Y. (2013). tranSMART: an open source and community-driven informatics and data sharing platform for clinical and translational research. AMIA Summ. Trans. Sci. Proc. 2013, 6–8. 24303286 PMC3814495

[B5] Barabáasi A.-L. Gulbahce N. Loscalzo J. (2011). Network medicine: a network-based approach to human disease. Nat. Rev. Genet. 12, 56–68. 10.1038/nrg2918 21164525 PMC3140052

[B6] Basso K. Margolin A. A. Stolovitzky G. Klein U. Dalla-Favera R. Califano A. (2005). Reverse engineering of regulatory networks in human b cells. Nat. Genet. 37, 382–390. 10.1038/ng1532 15778709

[B7] Ben-Hamo R. Efroni S. (2011). Gene expression and network-based analysis reveals a novel role for hsa-mir-9 and drug control over the p38 network in glioblastoma multiforme progression. Genome Med. 3:77. 10.1186/gm293 22122801 PMC3308032

[B8] Breiman L. (1996). Bagging predictors. Mach. Learn. 24, 123–140. 10.1007/BF00058655

[B9] Breiman L. (2001). Random forests. Mach. Learn. 45, 5–32. 10.1023/A:1010933404324

[B10] Brown K. R. Jurisica I. (2005). Online predicted human interaction database. Bioinformatics 21, 2076–2082. 10.1093/bioinformatics/bti273 15657099

[B11] Bussemaker H. J. Li H. Siggia E. D. (2001). Regulatory element detection using correlation with expression. Nat. Genet. 27, 167–171. 10.1038/84792 11175784

[B12] Butte A. Kohane I. (2000). Mutual information relevance networks: Functional genomic clustering using pairwise entropy measurements. Pacif. Symp. Biocomput. 5, 415–426. 10902190 10.1142/9789814447331_0040

[B13] Butte A. Tamayo P. Slonim D. Golub T. Kohane I. (2000). Discovering functional relationships between rna expression and chemotherapeutic susceptibility using relevance networks. Proc. Natl. Acad. Sci. U.S.A. 97, 12182–12186. 10.1073/pnas.220392197 11027309 PMC17315

[B14] Chan I. S. Ginsburg G. S. (2011). Personalized medicine: progress and promise. Ann. Rev. Genom. Hum. Genet. 12, 217–244. 10.1146/annurev-genom-082410-101446 21721939

[B15] Chen L. Xuan J. Riggins R. B. Clarke R. Wang Y. (2011). Identifying cancer biomarkers by network-constrained support vector machines. BMC Syst. Biol. 5:161. 10.1186/1752-0509-5-161 21992556 PMC3214162

[B16] Chuang H.-Y. Lee E. Liu Y.-T. Lee D. Ideker T. (2007). Network-based classification of breast cancer metastasis. Mol. Syst. Biol. 3, 140. 10.1038/msb4100180 17940530 PMC2063581

[B17] Dehmer M. Emmert-Streib F. (2007). Comparing large graphs efficiently by margins of feature vectors. Appl. Math. Comput. 188, 1699–1710. 10.1016/j.amc.2006.11.185

[B18] Dehmer M. Grabner M. Mowshowitz A. Emmert-Streib F. (2013c). An efficient heuristic approach to detecting graph isomorphism based on combinations of highly discriminating invariants. Adv. Comput. Math. 39, 311–325. 10.1007/s10444-012-9281-0

[B19] Dehmer M. Mehler A. (2007). A new method of measuring similarity for a special class of directed graphs. Tatra Mount. Math. Public. 36, 39–59

[B20] Dehmer M. Mueller L. Emmert-Streib F. (2013b). Quantitative network measures as biomarkers for classifying prostate cancer disease states: a systems approach to diagnostic biomarkers. PLoS ONE 8:e77602. 10.1371/journal.pone.0077602 24236006 PMC3827206

[B21] Dehmer M. Mueller L. A. J. Emmert-Streib F. (2013a). Quantitative network measures as biomarkers for classifying prostate cancer disease states: a systems approach to diagnostic biomarkers. PLoS ONE 8:e77602. 10.1371/journal.pone.0077602 24236006 PMC3827206

[B22] de Matos Simoes R. Dehmer M. Emmert-Streib F. (2013a). Interfacing cellular networks of *S. cerevisiae* and *E. coli*: Connecting dynamic and genetic information. BMC Genomics 14:324. 10.1186/1471-2164-14-324 23663484 PMC3698017

[B23] de Matos Simoes R. Dehmer M. Emmert-Streib F. (2013b). B-cell lymphoma gene regulatory networks: biological consistency among inference methods. Front. Genet. 4:281. 10.3389/fgene.2013.00281 24379827 PMC3864360

[B24] de Matos Simoes R. Emmert-Streib F. (2012). Bagging statistical network inference from large-scale gene expression data. PLoS ONE 7:e33624. 10.1371/journal.pone.0033624 22479422 PMC3316596

[B25] Emmert-Streib F. Altay G. (2010). Local network-based measures to assess the inferability of different regulatory networks. IET Syst. Biol. 4, 277–288. 10.1049/iet-syb.2010.0028 20632777

[B26] Emmert-Streib F. de Matos Simoes R. Mullan P. Haibe-Kains B. Dehmer M. (2014). The gene regulatory network for breast cancer: integrated regulatory landscape of cancer hallmarks. Front. Genet. 5:15. 10.3389/fgene.2014.00015 24550935 PMC3909882

[B27] Emmert-Streib F. Glazko G. (2011). Network biology: a direct approach to study biological function. Wiley Interdiscip. Rev. Syst. Biol. Med. 3, 379–391. 10.1002/wsbm.134 21197659

[B28] Emmert-Streib F. Glazko G. Altay G. de Matos Simoes R. (2012). Statistical inference and reverse engineering of gene regulatory networks from observational expression data. Front. Genet. 3:8. 10.3389/fgene.2012.00008 22408642 PMC3271232

[B29] Faith J. J. Hayete B. Thaden J. T. Mogno I. Wierzbowski J. Cottarel G.. (2007). Large-scale mapping and validation of *Escherichia coli* transcriptional regulation from a compendium of expression profiles. PLoS Biol. 5:e8. 10.1371/journal.pbio.0050008 17214507 PMC1764438

[B30] Friedman N. (2004). Inferring cellular networks using probabilistic graphical models. Science 303, 799–805. 10.1126/science.1094068 14764868

[B31] Frohlich H. Beissbarth T. Tresch A. Kostka D. Jacob J. Spang R.. (2008). Analyzing gene perturbation screens with nested effects models in R and bioconductor. Bioinformatics 24, 2549–2550. 10.1093/bioinformatics/btn446 18718939 PMC2732276

[B32] Fortney K. Xie W. Kotlyar M. Griesman J. Kotseruba Y. Jurisica I. (2013). NetwoRx: connecting drugs to networks and phenotypes in Saccharomyces cerevisiae. Nucleic Acids Res. 41, D720–D727. 10.1093/nar/gks1106 23203867 PMC3531049

[B33] Ghosh S. Basu A. (2012). Network medicine in drug design: implications for neuroinflammation. Drug Discov. Today 17, 600–607. 10.1016/j.drudis.2012.01.018 22326234

[B34] Guan Y. Gorenshteyn D. Burmeister M. Wong A. K. Schimenti J. C. Handel M. A.. (2012). Tissue-specific functional networks for prioritizing phenotype and disease genes. PLoS Comput. Biol. 8:e1002694. 10.1371/journal.pcbi.1002694 23028291 PMC3459891

[B35] Haibe-Kains B. Olsen C. Djebbari A. Bontempi G. Correll M. Bouton C.. (2012). Predictive networks: a flexible, open source, web application for integration and analysis of human gene networks. Nucleic Acids Res. 40, D866–D875. 10.1093/nar/gkr1050 22096235 PMC3245161

[B36] Hanahan D. Weinberg R. A. (2011). Hallmarks of cancer: the next generation. Cell 144, 646–674. 10.1016/j.cell.2011.02.013 21376230

[B37] Hecker M. Lambeck S. Toepfer S. van Someren E. Guthke R. (2009). Gene regulatory network inference: data integration in dynamic models - A review. Biosystems 96, 86–103. 10.1016/j.biosystems.2008.12.004 19150482

[B38] Huynh-Thu V. A. Irrthum A. Wehenkel L. Geurts P. (2010). Inferring regulatory networks from expression data using tree-based methods. PLoS ONE 5:e12776. 10.1371/journal.pone.0012776 20927193 PMC2946910

[B39] Ideker T. Krogan N. J. (2012). Differential network biology. Mol. Syst. Biol. 8, 565. 10.1038/msb.2011.99 22252388 PMC3296360

[B40] Islam M. Hoque M. Banik R. Roy S. Sumi S. Hassan F. M.. (2013). Comparative analysis of differential network modularity in tissue specific normal and cancer protein interaction networks. J. Clin. Bioinform. 3:19. 10.1186/2043-9113-3-19 24093757 PMC3852839

[B41] Kanehisa M. Goto S. (2000). KEGG: kyoto encyclopedia of genes and genomes. Nucleic Acids Res. 28, 27–30. 10.1093/nar/28.1.27 10592173 PMC102409

[B42] Lamb J. Crawford E. D. Peck D. Modell J. W. Blat I. C. Wrobel M. J.. (2006). The connectivity map: using gene-expression signatures to connect small molecules, genes, and disease. Science 313, 1929–1935. 10.1126/science.1132939 17008526

[B43] Lee W. P. Tzou W. S. (2009). Computational methods for discovering gene networks from expression data. Brief. Bioinform. 10, 408–423. 10.1093/bib/bbp028 19505889

[B44] Liang S. Fuhrman S. Somogyi R. (1998). Reveal, a general reverse engineering algorithm for inference of genetic network architectures. Pac. Symp. Biocomput. 1998, 18–29. 9697168

[B45] Luo W. Hankenson K. Woolf P. (2008). Learning transcriptional regulatory networks from high throughput gene expression data using continuous three-way mutual information. BMC Bioinformatics 9:467. 10.1186/1471-2105-9-467 18980677 PMC2613931

[B46] Madhamshettiwar P. Maetschke S. Davis M. Reverter A. Ragan M. (2012). Gene regulatory network inference: evaluation and application to ovarian cancer allows the prioritization of drug targets. Genome Med. 4:41. 10.1186/gm340 22548828 PMC3506907

[B47] Marbach D. Costello J. C. KÙffner R. Vega N. M. Prill R. J. Camacho D. M.. (2012). Wisdom of crowds for robust gene network inference. Nat. Methods 9, 796–804. 10.1038/nmeth.2016 22796662 PMC3512113

[B48] Margolin A. A. Nemenman I. Basso K. Wiggins C. Stolovitzky G. Dalla Favera R.. (2006). ARACNE: an algorithm for the reconstruction of gene regulatory networks in a mammalian cellular context. BMC Bioinformatics 7 Suppl. 1:S7. 10.1186/1471-2105-7-S1-S7 16723010 PMC1810318

[B49] Markowetz F. Spang R. (2007). Inferring cellular networks Đ a review. BMC Bioinformatics 8 Suppl. 6:S5. 10.1186/1471-2105-8-S6-S5 17903286 PMC1995541

[B50] Meyer P. Lafitte F. Bontempi G. (2008). minet: a R/Bioconductor package for inferring large transcriptional networks using mutual information. BMC Bioinformatics 9:461. 10.1186/1471-2105-9-461 18959772 PMC2630331

[B51] Mithani A. Hein J. Preston G. M. (2011). Comparative analysis of metabolic networks provides insight into the evolution of plant pathogenic and nonpathogenic lifestyles in pseudomonas. Mol. Biol. Evol. 28, 483–499. 10.1093/molbev/msq213 20709733

[B52] Mostafavi S. Ray D. Warde-Farley D. Grouios C. Morris Q. (2008). GeneMANIA: a real-time multiple association network integration algorithm for predicting gene function. Genome Biol. 9 Suppl. 1 S4. 10.1186/gb-2008-9-s1-s4 18613948 PMC2447538

[B53] Olsen C. Fleming K. Prendergast N. Rubio R. Emmert-Streib F. Bontempi G.. (2014). Inference and validation of predictive gene networks from biomedical literature and gene expression data. Genomics 103, 329–336. 10.1016/j.ygeno.2014.03.004 24691108 PMC4119824

[B54] Pache R. A. Aloy P. (2012). A novel framework for the comparative analysis of biological networks. PLoS ONE 7:e31220. 10.1371/journal.pone.0031220 22363585 PMC3283617

[B55] Przulj N. (2007). Biological network comparison using graphlet degree distribution. Bioinformatics 23, e177–e183. 10.1093/bioinformatics/btl301 17237089

[B56] Rozenblatt-Rosen O. Deo R. C. Padi M. Adelmant G. Calderwood M. A. Rolland T.. (2012). Interpreting cancer genomes using systematic host network perturbations by tumour virus proteins. Nature 487, 491–495. 10.1038/nature11288 22810586 PMC3408847

[B57] Schadt E. (2009). Molecular networks as sensors and drivers of common human diseases. Nature 461, 218–223. 10.1038/nature08454 19741703

[B58] Sharan R. Ideker T. (2006). Modeling cellular machinery through biological network comparison. Nat. Biotechnol. 24, 427–433. 10.1038/nbt1196 16601728

[B59] Shapira S. D. Gat-Viks I. Shum B. O. V. Dricot A. Grace M. M. D. Wu L.. (2009). A physical and regulatory map of Host-Influenza interactions reveals pathways in H1N1 infection. Cell 139, 1255–1267. 10.1016/j.cell.2009.12.018 20064372 PMC2892837

[B60] Slater T. (2014). Recent advances in modeling languages for pathway maps and computable biological networks. Drug Disco. Today 19, 193–198. 10.1016/j.drudis.2013.12.011 24444544

[B61] Staiger C. Cadot S. Kooter R. Dittrich M. Müller T. Klau G. W.. (2012). A critical evaluation of network and pathway-based classifiers for outcome prediction in breast cancer. PLoS ONE 7:e34796. 10.1371/journal.pone.0034796 22558100 PMC3338754

[B62] Stolovitzky G. Prill R. Califano A. (2009). Lessons from the DREAM 2 challenges. Ann. N.Y. Acad. Sci. 1158, 159–195. 10.1111/j.1749-6632.2009.04497.x 19348640

[B63] Werhli A. Grzegorczyk M. Husmeier D. (2006). Comparative evaluation of reverse engineering gene regulatory networks with relevance networks, graphical gaussian models and bayesian networks. Bioinformatics 22, 2523–2531. 10.1093/bioinformatics/btl391 16844710

[B64] Wille A. Zimmermann P. Vranova E. Furholz A. Laule O. Bleuler S.. (2004). Sparse graphical gaussian modeling of the isoprenoid gene network in *arabidopsis thaliana*. Genome Biol. 5:R92. 10.1186/gb-2004-5-11-r92 15535868 PMC545783

[B65] Yosef N. Shalek A. K. Gaublomme J. T. Jin H. Lee Y. Awasthi A.. (2013). Dynamic regulatory network controlling th17 cell differentiation. Nature 496, 461–468. 10.1038/nature11981 23467089 PMC3637864

[B66] Zhang H. Singer B. H. (2010). Recursive Partitioning and Applications (New York, NY: Springer). 10.1007/978-1-4419-6824-1

[B67] Zhang X. Zhao X.-M. He K. Lu L. Cao Y. Liu J.. (2011). Inferring gene regulatory networks from gene expression data by PC-algorithm based on conditional mutual information. Bioinformatics 28, 98–104. 10.1093/bioinformatics/btr626 22088843

